# DNA sequences performs as natural language processing by exploiting deep learning algorithm for the identification of N4-methylcytosine

**DOI:** 10.1038/s41598-020-80430-x

**Published:** 2021-01-08

**Authors:** Abdul Wahab, Hilal Tayara, Zhenyu Xuan, Kil To Chong

**Affiliations:** 1 Department of Electronics and Information Engineering, Jeonbuk National University, Jeonju, 54896 South Korea; 2 School of International Engineering and Science, Jeonbuk National University, Jeonju, 54896 South Korea; 3 Department of Biological Sciences, The University of Texas at Dallas, Richardson, 75080 USA; 4 Advanced Electronics and Information Research Center, Jeonbuk National University, Jeonju, 54896 South Korea

**Keywords:** Computational biology and bioinformatics, Computational models, Genome informatics, Machine learning, Software

## Abstract

N4-methylcytosine is a biochemical alteration of DNA that affects the genetic operations without modifying the DNA nucleotides such as gene expression, genomic imprinting, chromosome stability, and the development of the cell. In the proposed work, a computational model, 4mCNLP-Deep, used the word embedding approach as a vector formulation by exploiting deep learning based CNN algorithm to predict 4mC and non-4mC sites on the *C.elegans* genome dataset. Diversity of ranges employed for the experimental such as corpus k-mer and k-fold cross-validation to obtain the prevailing capabilities. The 4mCNLP-Deep outperform from the state-of-the-art predictor by achieving the results in five evaluation metrics by following; Accuracy (ACC) as 0.9354, Mathew’s correlation coefficient (MCC) as 0.8608, Specificity (Sp) as 0.89.96, Sensitivity (Sn) as 0.9563, and Area under curve (AUC) as 0.9731 by using 3-mer corpus word2vec and 3-fold cross-validation and attained the increment of 1.1%, 0.6%, 0.58%, 0.77%, and 4.89%, respectively. At last, we developed the online webserver http://nsclbio.jbnu.ac.kr/tools/4mCNLP-Deep/, for the experimental researchers to get the results easily.

## Introduction

DNA methylation is a mechanism that entails the chemical modification of DNA sequences, that changes hereditary performance without altering the DNA’s nucleobases. DNA modification through methylation and demethylation plays a significant role in gene expression. DNA methylation can regulate various biological processes including genomic imprinting, chromosome stability, and cell development and extend the assortment of genes because of its structural changes in DNA^1^. Prokaryotic and eukaryotic genomes undergo three types of methylation; N4-methylcytosine (4mC)^2^, 5-Methylcytosine (5mC)^3^, and N6-methyladenine (6mA)^4^.

Gene modifications are assembled by the distinct DNA methyltransferases (DNMTs) to transmit a methyl group to a particular exocyclic amino group^5^. 5mC is one of the most extensively studied types of cytosine methylation as a consequence of its widespread dissemination and complicated aspects^6^. 5mC plays an important role in numerous biological processes^7^ associated with neurological diseases, diabetes, and cancer. 6mA, in contrast, takes place only on a very small-scale, and is only found in eukaryotes using high sensitivity methods. 4mC is considered a dynamic epigenetic modification because of the restriction-modification (R-M) method to protect restriction enzyme form deterioration of self-DNA. It was first discovered in 1983^8^. 4mC plays a significant role in the regulation of a number of processes intrinsic to the cell cycle, including gene expression, defining self and non-self-DNA , DNA replication, and correcting DNA replication errors^9,10^. Investigational studies related to 4mC have waned in part due to a lack of sufficient identification techniques. While there are several experimental procedures capable of identifying 4mC sites, including mass spectrometry, for the whole genome 4mC-Tet-assisted bisulfite, Single-Molecule of Real-Time (SMRT) sequencing, and methylation-precise PCR^11–14^, these approaches are regarded as expensive and time-consuming when applied across an entire genome.

In recent years, various novel computational N4-methylcytosine site identification methods have been proposed and applied across a diverse number of species, including *Geoalkalibacter subterraneus*, *Arabidopsis thaliana*, *Geobacter pickeringii*, *Escherichia coli*, *Drosophila melanogaster*, and *Caenorhabditis elegans*^15–19^. These methods rely on state-of-the-art machine learning (ML) algorithms to make their predictions for 4mC sites. Each of these predictors used different encoding techniques such as binary encoding, nucleotide chemical properties, and nucleotide frequencies with various algorithms like support vector machine, random forest, and decision tree. Recently, a new predictor DNC4mC-Deep^20^ was proposed to identify and analyze of Rosaceae genome, where they implemented a deep learning based Convolution Neural Network (CNN) algorithm. They used six different kinds of encoding methods binary encoding (BE), dinucleotide composition (DNC), trinucleotide composition (TNC), Nucleotide chemical property (NCP), nucleotide chemical property and nucleotide frequency (NCPNF), and multivariate mutual information (MMI). Another deep learning based model has been established named as 4mCDeep-CBI^21^ to identify the N4-methylcytosine sites in the newly developed dataset of Caenorhabditis elegans, where they implemented 3-CNN and Bidirectional Long Short-Term Memory (BLSTM) to fetch the deep features for the prediction.

In this work, we developed a new tool, named 4mCNLP-Deep, to identify and analyze 4mC sites associated with *C. elegans* dataset which was recently expanded by increasing the number of samples. The Structure of the proposed model was built as follows; First, we used the encoding method word2vec, which has never been used before in N4-methylcytosine identification, to transform sequences into vectors form using word embedding. The word-embedding approach mostly operates on Natural Language Processing (NLP)^22^, but thereafter executed efficaciously on wide-genome identification^23–28^. We obtained the final CNN model by applying the grid search algorithm with tuned hyper-parameters and fed the vectors of word embedding into it. We used a K-fold cross-validation method for different values of K. Then, we applied five evaluation metrics to assess the model. We also employed two applications, silico mutagenesis^29^ and saliency^30^ map to interpret the predictive deep learning model and influence of important features. The results of the predictive model showed outperformance when compared to the state-of-the-art model. 4mCNLP-Deep successfully achieves 0.9354, 0.8608, 0.8996, 09563, and 0.9731 for Accuracy (ACC), Mathew’s correlation coefficient (MCC), Sensitivity (Sn), Specificity (Sp), and Area under the curve (AUC), respectively on C. elegan dataset by using 3-mer corpus word2vec and 3-fold cross-validation. Our model attained the increment of 1.1% on ACC, 0.6% on MCC, 0.58% on Sn, 0.77% Sp, and 4.89% on AUC, respectively.

## Materials and methods

### Benchmark datasets

The benchmark dataset of *Caenorhabditis elegans* (*C. elegans*) was attained from Feng Zeng et al.^21^. Where they extend the existing dataset of Ye et al.^31^ by producing new samples. New samples were got from the MethSMRT database consisted of 4mC and non 4mC sites, where each had a length of 41 bp. Two steps were taken, first, in the Methylome Analysis Technical Note, it was shown that the modification QV (modQV) score for the IPD ratio had remarkably dissimilar from the estimated. With the modQV score of greater than 30, the samples were removed. Next, they used the CD-HIT^32^ software to remove the redundancy of bias samples to make sure a biased dataset will not miscalculate the accuracy results. The cut off frequency was used 0.80.

Subsequently, newly acquired samples were integrated with the benchmark dataset which was used in several research works. A dataset formed with the number of 18747 samples, to reduce the similarity between the new and old samples a CD-HIT was used. After that, a new dataset of *C. elegans* prepared with a total of 17808 samples from which 11173 are 4mC samples and 6635 are non 4mC samples.

**Table 1:** Parameters of word2vec model which were used in training.

Parameters	Word2vec learning model
Training approach	CBOW
Corpus	*C. elegans* (WBcel235)
Context words	(2-mer, 3-mer, 4-mer)
Vector size	100
Minimum count	5
Negative sampling	5
Window size	5
Number of epochs	20

### Distributed feature representation

The nature of raw genomic datasets is considered as complicated and noisy. With this necessity, we focused to apply the computational model for the instinctive feature representation learning approach on genomic data^33^. This method allows for inducing optimum features set and increases the performance of the computational model by reducing the model complexity.

Vector representation of words or word embedding is the most well-known technique in the natural language processing (NLP) operations. Theoretically, it transforms the 1-dimension per word into continuous N-dimension vectors. The first word2vec model was proposed by Mikolov et al.^22^ Based on a neural network, resultant outcomes possessed distributed characterize sentences of linguistic words. The aforementioned, technique was much faster than the preceding methods, to train the model for continuous vector space and lower the dimensions. In recent years, the success of NPL has been shown caused by its advantageous applications for instance speech recognition, language assist, and translation devices, which made substantial progression on the word embedding methods. Furthermore, researchers revealed that genetic data can be used as language whether DNA or RNA samples that occur within the structure of the cell^34–36^. Additionally, several biological related complex problems have been successfully demonstrated through NLP approaches^25–28,37^.

Corpus development is the first step for implementing the word2vec model, it divides the continuous biological sequence into k length (k-mer)^38^ of nucleotides groups to formulate as a word and identify linguistic associations among them. In this work, we have carried out the preprocessing to compose the text corpus from the *C. elegans* genome and then trained the word2vec model. We have produced the corpus by operating the whole *C. elegans* genome assembly (WBcel235/ce11) which is downloaded from http://hgdownload.soe.ucsc.edu/goldenPath/ce11/chromosomes/.

Firstly, the genome assembly was distributed into seven chromosomes (chrI, chrII, chrIII, chrIV, chrM, chrV, chrX) and then each chromosome split into the sequence of 41nt to shape the sentence. A continuous bag-of-words (CBOW) approach has been employed to train the word2vec model. CBOW determines the recent word *w*(*t*) by surrounds the context word based on predefined window size which was set as 5. A biological sequence which is mostly a combination of A, C, G, T nucleotides transforms into the sequence of words by setting the k-mer value. The k was set k = 3 with overlapping which forms a DNA sequence ACGTCAGT into words like ACG, CGT, GTC, TCA, CAG, AGT. Each 3-mer word is indicated by a 100-dimensional vector. We experimented with the word2vec model by different values of k-mer, such as k=2, k=3, k=4. Complete details of the parameters which were used are shown in Table 1.

## The proposed model

In this work, adequate deep learning based CNN model was proposed for the prediction of N4-methylcytosine sites of the *C. elegans* genome. CNN has the capability to acquire leading quality features automatically for the classification prediction instead of manually handy crafted like traditional supervised learning methods. Whereas, an assorted CNN model can be made by using handcrafted features. Convolution Neural Network has been utilized in several research areas such as image processing^39,40^, natural language processing^41^, and computational biology^42–47^. A grid search algorithm was implemented with different hyper-parameters values to obtain the most optimal CNN model during its learning. The range of parameters is demonstrated in Table 2.

**Table 2:** Demonstration of hyper-parameter tuning of proposed model.

Parameters	Range
Number of convolution layers	[1,2,3,4,5]
Filters in convolution layer	[8, 10, 12, 16, 18, 25, 32, 44, 64, 128]
Filter size	[2, 3, 4, 5, 6, 8, 10, 12]
Number of groups in GroupNormalization layer	[2, 4]
Pool-size in maxpooling layer	[2, 4]
Stride length in maxpooling	[2, 4]
Dropout values	[0.15, 0.2, 0.25, 0.3, 0.35, 0.4, 0.45]

**Figure 1:**
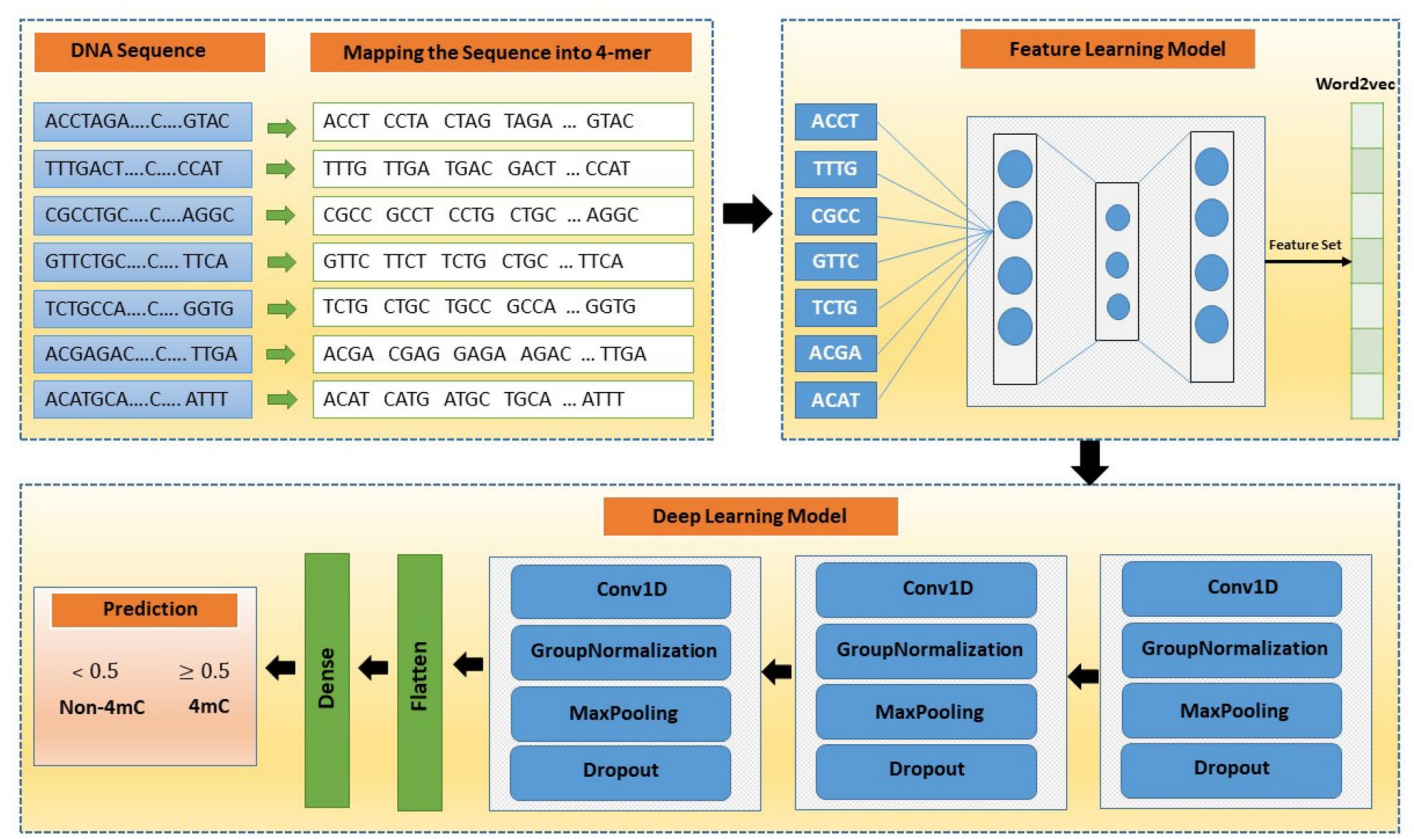
Illustration of complete architecture of 4mCNLP-Deep.

In the proposed work, the word2vec feature representation was introduced which is completely different from previous works of N4-methylcytosine. A CNN based word2vec model trained on the optimum model which got from the grid search. The input of the model is $(L - k + 1) \times 100$, where L is the length of the input sequence, k is the value of k-mer and 100 is a dimensional vector for each word in the sequence sentence. The model contains three blocks and each has several layers with diverse parameter ranges to construct the model. Each block comprises a convolution layer (Conv1D) having parameters as a filter number with values of 32, 32, 16, respectively, kernel-size with values of 5, 5, 4, correspondingly and stride with the value of 1 for all. The convolution layer has capability to extract the features by self-activating for the appropriate input. In all the convolution layers, L2 regularization weights and bias used to assure the model by overfitting. The values of both regularizations were set as 0.0001, for all three Conv1D, an exponential linear unit (ELU) utilized as an activation function. Each block Conv1D was followed by a group normalization layer (GN), which condensed the consequences of convolution layers. GN distributes the feature map into the desired numbers of groups and normalizes them within each group. The number of groups was fixed as 4, 4, 2, respectively in each block. Moreover, a max-pooling layer (MaxPooling1D) was applied to minimize the dispensable features after the GN layer and avail to turn down the dimensionality of the features. The pool-sizes were set as 4, 4, 2, correspondingly, and strides were set as 2 for all max-pooling layers in each block. Right after MaxPooling1D, dropout layers were used to avoid the overfitting problem while training the model. It supports by turning off the operations of some hidden nodes by regulating the neurons to zero at the learning process.

After the convolution blocks, a flatten layer was used to unstack the outcomes and squash the features vectors from preceding layers. Furthermore, a fully connected layer (FC) was implemented with the number of 32 neurons and L2 weights and bias regularization was utilized by setting the value at 0.001. In the FC layer, ELU activation was used. Finally, the last FC layer was employed with a sigmoid activation function for the bimodal classification. The sigmoid function helps to squeeze the outcome numbers on the scale of between 0 and 1 and demonstrates the likelihood of acquiring the 4mC and non-4mC sites. Figure 1 shows the detailed architecture of presented CNN model and feature learning model.

The proposed model 4mCNLP-Deep was executed on Keras^48^. Stochastic gradient descent (SGD) optimizer was used with the value of 0.95 for momentum and 0.004 for the learning rate. Binary cross-entropy is deployed as a loss function. We fix the 100 epochs and 32 batch size for the fit function. The call back function was used to storing models and their corresponding weights by calling the checkpoint. While early stopping is used to stops the prediction accuracy at a certain point once validation puts an end to improve. The early stopping patience level was set as 20.

## Performance evaluation metrics

The effective performance of the 4mCNLP-Deep model was measured by k-fold cross-validation, we used three different values for the k such as 3 fold, 5 fold, and 10 fold cross-validation to carry out the preeminent identification. Cross-validation is used to estimate the explicit achievement of the desired model by using the resampling method. The whole dataset merges and splits into k number of clusters, each cluster carries eight folds for training, one for validation, one for testing. The proposed CNN model was trained and tested k intervals. There are four metrics to evaluate the performance of the model such as Accuracy (ACC), Mathew’s correlation coefficient (MCC), Specificity (Sp), and Sensitivity (Sn) with the given mathematical formulation^49–51^.

1 $$\begin{aligned} ACC= & {} \frac{TP + TN}{TP + TN + FP + FN} \end{aligned}$$

2 $$\begin{aligned} MCC= & {} \frac{ TP * TN - FP * FN}{\sqrt{(TP + FP)*(TP + FN)*(TN + FP)*(TN + FN)}} \end{aligned}$$

3 $$\begin{aligned} SP= & {} \frac{TN}{TN + FP} \end{aligned}$$

4 $$\begin{aligned} SN= & {} \frac{TP}{TP + FN} \end{aligned}$$

Where TN and TP represent as true negative and true positive having the correct number of identified sequences related to 4mC and non-4mC, respectively. Whereas, FN and FP denote as false negative and false positive taking false number of identified sequences for 4mC and non-4mC, respectively. Moreover, the receiver operating characteristics curve (ROC) and area under the ROC curve (AUC) were also deployed to demonstrate the achievement of the presented deep learning model.

**Table 3:** Results demonstration of 4mCNLP-Deep with different experimental values compare to state-of-the-art model on benchmark dataset for *C. elegans*.

Value of k-mer	No. of folds	ACC	MCC	Sp	Sn	AUC
4mCNLP-Deep (2-mer)	3-fold	0.9323	0.8542	0.8973	0.9528	0.9730
	5-fold	0.9343	0.8585	0.9027	0.9528	0.9742
	10-fold	0.9382	0.8668	0.9034	0.9586	0.9758
4mCNLP-Deep (3-mer)	3-fold	0.9354	0.8608	0.8996	0.9563	0.9731
	5-fold	0.9427	0.8766	0.9141	0.9595	0.9764
	10-fold	0.9455	0.8825	0.9132	0.9643	0.9788
4mCNLP-Deep (4-mer)	3-fold	0.9328	0.8551	0.8974	0.9535	0.9732
	5-fold	0.9446	0.8805	0.9097	0.9650	0.9768
	10-fold	0.9500	0.8922	0.9209	0.9670	0.9798
4mC-Deep CBI	3-fold	0.9294	0.8498	0.8938	0.9486	0.9242

## Results and discussion

A word2vec formulation technique was utilized with different ranges of k-fold cross-validation to predict the N4-methylcytosine by the implemented an optimal predictor to obtain the best performance.

### Performance evaluation

In the proposed model 4mCNLP-Deep, we did diverse experiments with a distinct assortment of values for corpus k-mer (2, 3, 4) and k-fold (3, 5, 10) on the *C. elegan* dataset. Each model utilized word2vec with different k-mer values on a various number of folds to check the best performance. For example 2-mer word2vec was implemented with distinct folds of 3, 5, and 10 as cross-validation. As the value of k increases, the disparity in size among the training set and the resampling subset gets shorter. In the resulting model returns immeasurable results. If the difference increase, the bias of the procedure becomes larger and it affects the model outcomes as compared to a large difference. In contrast, the proposed model gives better results in 10 folds of each k-mer of word2vec.

**Figure 2:**
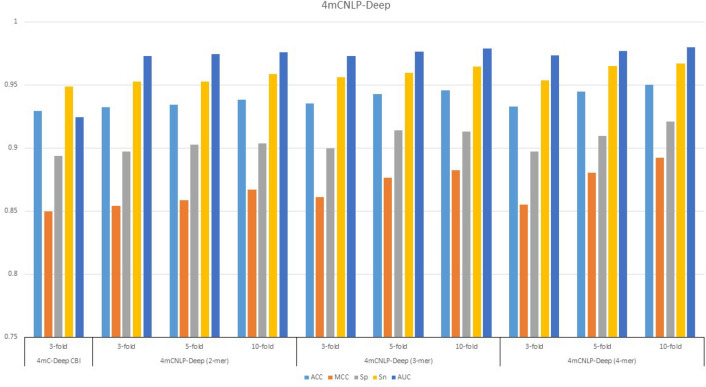
Depiction of performance comparison of 4mCNLP-Deep and existing model with various experimental values.

For constructive comparison, we compared our predictor with state-of-the-art model 4mCDeep-CBI^21^ and scrutinize the credibility of the model to identify the 4mC and non-4mC sites. The 4mcDeep-CBI applied 3-fold, our model outperformed with 3-mer word2vec using 3-fold and has reported as ACC of 0.93.54, MCC of 0.8608, Sp of 0.89.96, Sn of 0.9563, and AUC of 0.9731 and accomplished increment of 1.1%, 0.6%, 0.58%, 0.77%, and 4.89%, respectively. The detailed experimental results of 4mCNLP-Deep with all ranges have been shown in Table 3. The performance evaluation of 4mCNLP-Deep and state-of-the-art are demonstrated in Fig. 2.

**Figure 3:**
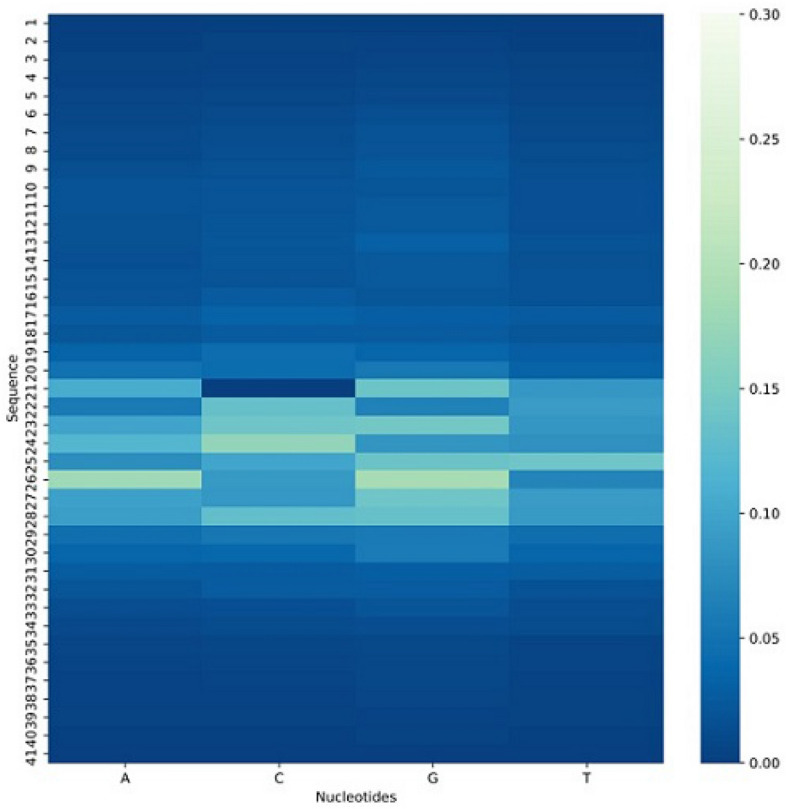
Visualization of heatmap of silico mutation, Where the C nucleotide in the center shows the weak effect on the final prediction as compare to other nucleotides.

### Applications for interpreting deep learning model

Deep learning models have the capability to attain the pioneering results but it’s laborious to interpret the algorithms as a standard statistical model. In the present study, there are two application established to understand and analyze the deep learning model by visualization techniques.

The first method to decipher a convolution neural network model for computational and statistical biology is silico mutagenesis which was used in various scientific works^29,52^. It is operated by mutating each nucleotide by a single base of sequences with a fixed length of four nucleotide A, C, G, T. In this systematical methodology, the model restore each outcome of resulted mutation and keeps the output as an absolute difference. Further, taken out the aggregated average of mutated predictions for the complete dataset.

For the mutated alterations, a heat map was implemented to show the impact of mutation. Figure 3, demonstrates the visualization of the mutation on the C. elegans dataset as an indigenous feature during the model’s learning phase. As it can be shown that the influence of mutation is less in the center of sequence on ultimate identification due to C nucleotide which is symbolized as N4-methylcytosine modification. The recasting of C nucleobase can intimate the unique kind of gene modification. In comparison, more influence of mutation can be shown on the other sides of the heat map which leads to represent the modification of nucleotides can change the results of cytosine recognition.

**Figure 4:**
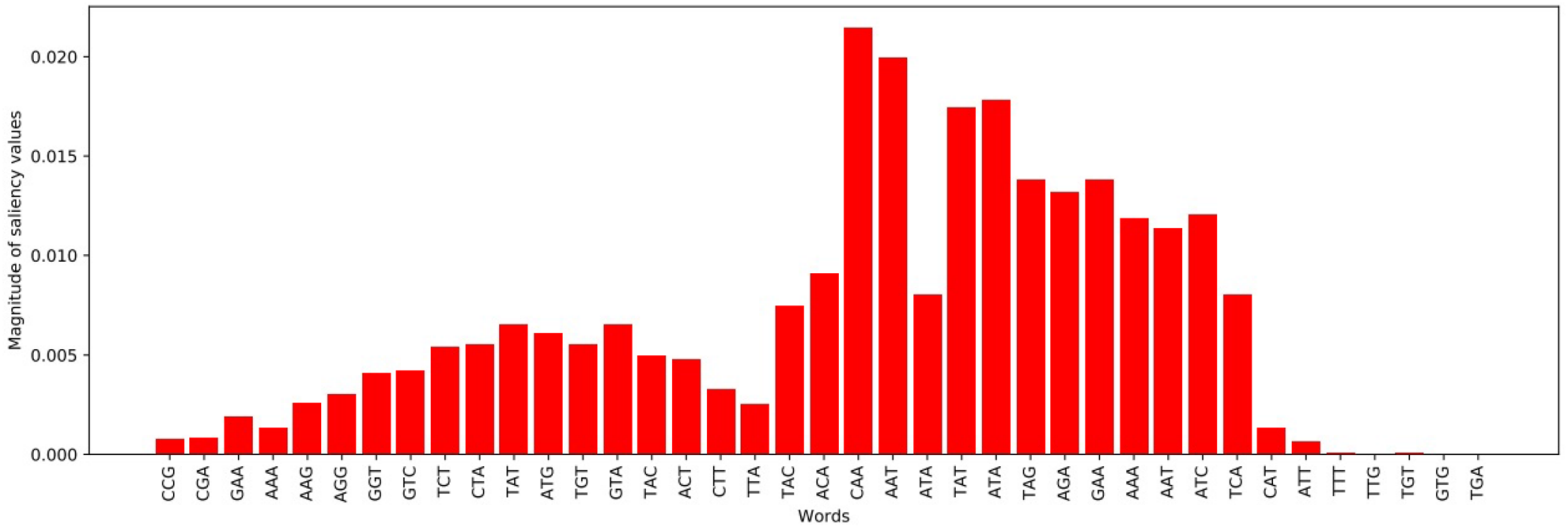
Illustration of saliency map of each 3-mer words having significant importance on the model outcome.

The second technique to interpret the deep learning based CNN model is the saliency map which helps to identify the most influential features of the sequence by the help of the gradient of the model for final prediction. It points out the most significant characteristics in the samples to classify the class related to the modification, several investigators used in their work^30,53^. For the envision, the efficiency of each location was derived by pointwise product of the saliency map through the vector encoding to obtain the imitative values of actual nucleotide characters of sequences such as A, C, G, T. We experimented by splitting the samples into 3-mer chunks across all the sequence by the formulation of $L-K+1$. The effect of tri-nucleotide letters at each place of the whole *C. elegans* dataset’s outcome result can be shown in Figure 4. In the middle of the words, the CAA motif has a significant magnitude value which is illustrating the utmost vital features in the sample for the identification of the CNN model. The base 4mCNLP-Deep also specifying the current gene modification related to N4-methylcytosine.

### Application of clinical research

*C. elegans* consider as a hereditary model organism used in the study of physiology, to sum up, the aspects of human disease. It is a widely applied non-mammalian animal model that is well proven for the highly versatile experiments for research on genetics, development, aging, muscle physiology, and radiobiology. The main purpose of the clinical research on *C. elegans* is to identify such type of genes which provides information about the mechanism of human disease development and also helps to enhanced diagnosis and treatment. Clinical experiments are costly and time-consuming when used for the whole genome. Therefore the computational approaches are utilized for several experiments that reduce the value and time. The presented model is made by considering this necessity, to ease clinical experimental biologist. They can easily detect the N4-methylcytosine site and then used further for the development of human disease identification or treatment. The proposed method already proved and contributing to the detection of different genetic sites in the genome, word2vec, and CNN made a big impact and utilized by several investigators^25–28,37^ to contribute as a better solution. This type of applications helps the biologist by freely accessible online tools.

## Conclusion

In the presented work, we introduced a persuasive computational biological model which is known as 4mCNLP-Deep for the prediction of 4mC and non-4mC sites. The expanded dataset of *C. elegans* was utilized for training and testing the deep learning model. Furthermore, a unique encoding technique was applied to transform sequences into the vectors representation by using a word embedding for the deep learning model. An optimal CNN algorithm was deployed after getting the best settings by exploiting hyperparameter tuning in a grid search. We performed several experiments for the values of k-mer in corpus and cross-validation for k-fold. All the experimental results are outperforming from the existed model. However, for rational comparison, 3-mer word2vec on 3-fold cross-validation has shown a prominent result which indicates the effective performance and high intelligence of the model for predicting the N4-methylcytosine sites. In the proposed work, five evaluation metrics were used like ACC, MCC, Sp, Sn, and AUC to measure the robustness and productivity of identification. Lastly, two diverse approaches named silico mutagenesis and saliency map were employed to interpret our deep learning based CNN model and understand the biological significance of gene modification. 4mCNLP-Deep can be appropriated by the biologists and create a high impact to identify a different kind of gene modification specifically N4-methylcytosine and specify the brain related diseases or development irregularities. In the future, we will expand the model complexity with a proper and efficient way for the prediction of all kinds of gene modification which will make a huge contribution in the field of bioinformatics and computational biology. Moreover, we developed the online webserver http://nsclbio.jbnu.ac.kr/tools/4mCNLP-Deep/, for the experimental researchers to get the results easily.
